# Hydrophilic Modification of Dialysis Membranes Sustains Middle Molecule Removal and Filtration Characteristics

**DOI:** 10.3390/membranes14040083

**Published:** 2024-04-03

**Authors:** Adam M. Zawada, Karlee Emal, Eva Förster, Saeedeh Saremi, Dirk Delinski, Lukas Theis, Florian Küng, Wenhao Xie, Joanie Werner, Manuela Stauss-Grabo, Matthias Faust, Skyler Boyington, James P. Kennedy

**Affiliations:** 1 Product Development, Fresenius Medical Care Deutschland GmbH, 66606 Sankt Wendel, Germany; eva.foerster@freseniusmedicalcare.com (E.F.); saeedeh.saremi@htwsaar.de (S.S.); dirk.delinski@freseniusmedicalcare.com (D.D.); lukas.theis@freseniusmedicalcare.com (L.T.); florian.kueng@freseniusmedicalcare.com (F.K.); 2 Applications Laboratory, Fresenius Medical Care, Ogden, UT 84404, USA; karlee.emal@freseniusmedicalcare.com (K.E.); skyler.boyington@freseniusmedicalcare.com (S.B.); 3 Institute for Physical Process Technology, Saarland University of Applied Sciences, 66117 Saarbrücken, Germany; matthias.faust@htwsaar.de; 4 Product Development, Fresenius Medical Care, Shanghai 200233, China; wenhao.xie@freseniusmedicalcare.com; 5 Clinical Marketing & Innovations, Fresenius Medical Care, Waltham, MA 02451, USA; joanie.werner@freseniusmedicalcare.com; 6 Global Biomedical Evidence Generation, Fresenius Medical Care Deutschland GmbH, 61352 Bad Homburg, Germany; manuela.stauss-grabo1@freseniusmedicalcare.com; 7 Product Development, Fresenius Medical Care, Ogden, UT 84404, USA; james.kennedy@freseniusmedicalcare.com

**Keywords:** clearance, performance, fouling, ultrafiltration, hemodiafiltration

## Abstract

While efficient removal of uremic toxins and accumulated water is pivotal for the well-being of dialysis patients, protein adsorption to the dialyzer membrane reduces the performance of a dialyzer. Hydrophilic membrane modification with polyvinylpyrrolidone (PVP) has been shown to reduce protein adsorption and to stabilize membrane permeability. In this study we compared middle molecule clearance and filtration performance of nine polysulfone-, polyethersulfone-, and cellulose-based dialyzers over time. Protein adsorption was simulated in recirculation experiments, while β2-microglobulin clearance as well as transmembrane pressure (TMP) and filtrate flow were determined over time. The results of this study showed that β2-microglobulin clearance (−7.2 mL/min/m^2^) and filtrate flow (−54.4 mL/min) decreased strongly during the first 30 min and slowly afterwards (−0.7 mL/min/m^2^ and −6.8 mL/min, respectively, for the next 30 min); the TMP increase (+37.2 mmHg and +8.6 mmHg, respectively) showed comparable kinetics. Across all tested dialyzers, the dialyzer with a hydrophilic modified membrane (FX CorAL) had the highest β2-microglobulin clearance after protein fouling and the most stable filtration characteristics. In conclusion, hydrophilic membrane modification with PVP stabilizes the removal capacity of middle molecules and filtration performance over time. Such dialyzers may have benefits during hemodiafiltration treatments which aim to achieve high exchange volumes.

## 1. Introduction

Most patients with end-stage renal disease (ESRD) depend on extracorporeal renal replacement therapies, such as hemodialysis (HD) or hemodiafiltration (HDF), to remove excess water and uremic toxins which accumulate due to the loss of kidney function. Importantly, many uremic toxins have been associated with ESRD-related comorbidities, such as cardiovascular disease, malnutrition, or inflammation [1,2,3,4,5]. Here, middle molecules, such as β2-microglobulin (~12 kDa), play a central role and have been linked to reduced survival rates in dialysis patients [2,6,7].

The recently published CONVINCE trial demonstrated a survival benefit for patients treated with high-volume HDF (convection volume ≥ 23 L) as compared to conventional HD [8], supporting the importance of middle molecule removal during dialysis treatment, as this has been discussed as a central element for the positive observations [1]. Importantly, the clearance of such molecules is directly correlated with the achieved ultrafiltration volume during treatment [1,9,10].

To achieve the treatment targets that were shown during the CONVINCE trial to impact patient outcomes, the dialysis session must be performed with a dialyzer that enables high ultrafiltration volumes and high middle molecule removal [11]. Dialyzer performance is mainly determined by the dialyzer membrane, which may differ in material composition, geometry, or structure, including the pore size and properties of the blood-facing surface [12,13,14,15,16,17,18,19]. Different dialyzers for the treatment of dialysis patients are available on the market, consisting of synthetic (e.g., polysulfone, polyethersulfone, polyacrylonitrile (PAN), polymethylmethacrylate (PMMA), polyester polymer alloy (PEPA), or ethylene-vinyl alcohol co-polymer (EVAL)) or cellulose-based membranes, which may differ in their physical properties, such as in terms of hydrophilicity, membrane potential, or permeability [13,14,15,16,17,20,21,22,23]. Most common dialyzers used in the clinical routine contain synthetic membranes based on polysulfone or polyethersulfone.

While the performance of dialyzers is mainly described by aqueous clearances in the respective instructions for use, it is important to note that the performance of a dialyzer is not a constant value but decreases over treatment time, especially during the first 30 min of treatment [17,19,24,25,26,27,28,29]. This reduction in performance is mainly caused by the contact and adsorption of plasma proteins to the membrane, leading to the buildup of a protein layer on the inner membrane surface, which provides an additional resistance for uremic toxin removal. This may necessitate more pressure on the membrane to achieve the same membrane flux as at the start of the treatment [12,19]. Such decreased permeability of the membrane is especially critical in the context of high-volume HDF, as this—together with the transmembrane pressure (TMP)—are two major determinants for the achieved ultrafiltration volume during HDF treatment.

To reduce protein adsorption to the membrane, recent advances in dialyzer technology have led to the development of synthetic high-flux membranes with increased amounts of the hydrophilic agent polyvinylpyrrolidone (PVP) on their blood-side surface [12,30,31,32,33,34,35,36]. The novel FX CorAL dialyzer contains such a hydrophilic membrane with increased PVP content on the blood-side surface and was shown in clinical and experimental studies to induce lower protein adsorption than other commonly used dialyzers in clinical practice [12,36,37,38]. Moreover, the FX CorAL dialyzer was previously compared to other polysulfone-, polyethersulfone-, and cellulose-based dialyzers with regard to performance and hemocompatibility [11,12,19,36,37,38,39,40,41]. Experimental studies showed a strong correlation between the low protein adsorption by the FX CorAL and the good hemocompatibility profile as well as low sieving coefficient changes over time [19,38]. In clinical studies, the FX CorAL consistently showed the highest β2-microglobulin removal rates and low activation of important hemocompatibility markers, such as complement activation, when compared to other polysulfone-, polyethersulfone-, and cellulose-based dialyzers [37,39,40,41].

The present in vitro study investigates for the first time how hydrophilic membrane modification, as implemented in the novel FX CorAL dialyzer, may help to sustain the clearance of the clinically relevant middle molecule β2-microglobulin and the filtration characteristics after protein adsorption to the membrane. The results of the present study may help to better understand how modifications in dialysis membranes could contribute to improved treatment of patients.

## 2. Materials and Methods

### 2.1. Investigated Dialyzers

The FX CorAL dialyzer (Fresenius Medical Care) was compared to eight further dialyzers, containing synthetic (polysulfone- or polyethersulfone-based) or cellulose-based membranes. Further information is provided in Table 1.

### 2.2. Determination of Middle Molecule Clearance after Protein Adsorption

**Figure 1:**
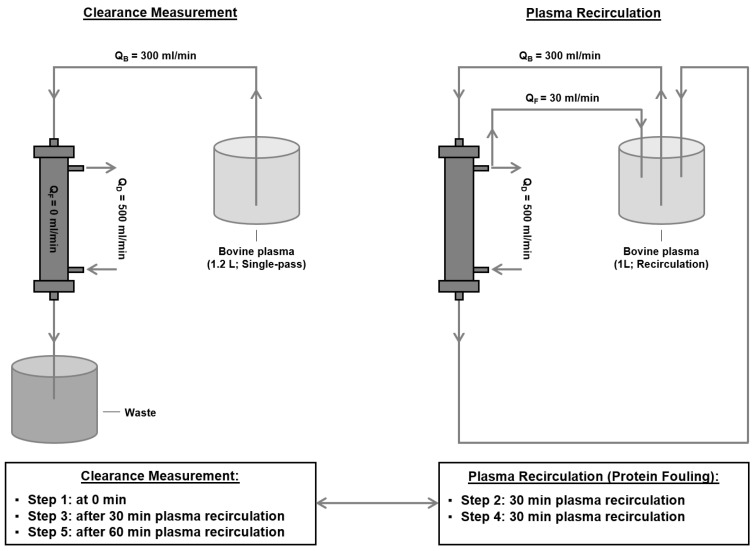
Setup to determine middle molecule clearance after protein adsorption. A two-step approach was used. (1) **Left**: Determination of β2-microglobulin clearance in a single-pass mode with bovine plasma at three different timepoints (0 min, 30 min, and 60 min after protein fouling); (2) **Right**: Induction of protein adsorption to the membrane in a recirculation system with bovine plasma; recirculation was performed before the second (30 min) and the third (60 min) clearance measurements, respectively.

To investigate the impact of protein adsorption on middle molecule clearance, a two-step approach was applied (Figure 1).

(1) *Determination of β2-microglobulin clearance:*

This test setup was performed using the 2008T hemodialysis machine from Fresenius Medical Care. Each dialyzer was first primed with isotonic sodium chloride solution (Fresenius Medical Care). Next, 1.2 L of bovine plasma (37 °C, 6.0 ± 0.5 g/dL total protein concentration, 1% sodium heparin, Lampire Biological Laboratories), spiked with 2.25 mg/L β2-microglobulin, 0.3 g/L inulin, 150 mg/L creatinine, and 1.9 g/L urea, was run in a single-pass mode through the dialyzer at a flow rate of 300 mL/min. The dialysate flow rate was 500 mL/min using NaturaLyte (2 K, 2.5 Ca, 1 Mg) and bicarbonate dialysate (Fresenius Medical Care). Plasma samples were taken before and after the dialyzer, and the concentrations of β2-microglobulin, inulin, creatinine, and urea were determined using an immunoturbidimetric assay for clinical chemistry analyzers (Roche/Hitachi), a colorimetric carbohydrate quantification method (in-house), an enzymatic creatininase clinical chemistry assay (Roche/Hitachi), and a kinetic blood urea nitrogen clinical chemistry assay (Roche/Hitachi), respectively. The study focuses on β2-microglobulin; concentrations of lower molecular weight parameters inulin (5 kDa), creatinine (113 Da), and urea (60 Da) were determined for comparison purposes. Each plasma sample was analyzed in triplicate for each analyte. Based on the mean analyte concentration and the blood flow rate, the respective clearances were determined according to the following formula:

(1) $$\;\mathrm{Clearance}=\left(1-\frac{Concentration\;post\;dialyzer}{Concentration\;pre\;dialyzer}\right)\cdot Blood\;flow$$

The clearance values were normalized based on the membrane surface size. Each dialyzer type was tested three times at three different time points (0 min, after 30 min of protein fouling, and after 60 min of protein fouling; see step 2).

(2) *Induction of protein adsorption to the membrane:*

This setup was also performed on the 2008T hemodialysis machine. After the initial clearance measurement (step 1), the respective dialyzer was used for this protein adsorption setup. Therefore, 1 L of bovine plasma was recirculated through the dialyzer at a blood flow rate (Q_B_) of 300 mL/min, filtrate flow rate (Q_F_) of 30 mL/min, and dialysate flow rate (Q_D_) of 500 mL/min. This recirculation was performed for 30 min after the first clearance measurement and again for 30 min after the second clearance measurement.

### 2.3. Characterization of Filtration Performance after Protein Adsorption

**Figure 2:**
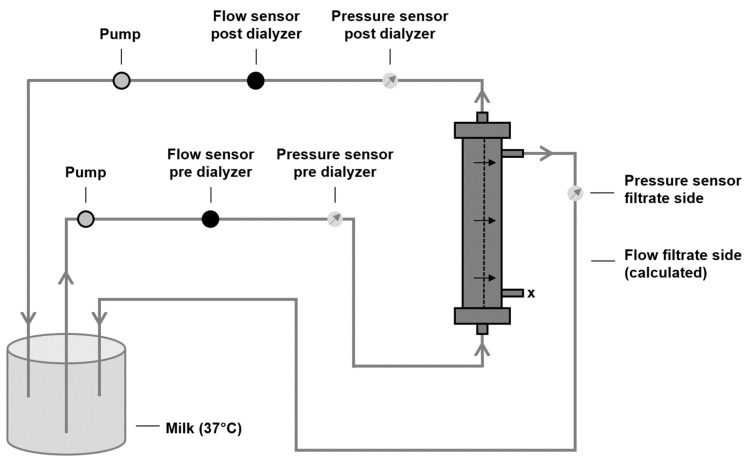
Setup to characterize filtration performance after protein adsorption. In a recirculation experiment, the increase in TMP at constant flow conditions as well as the decrease in filtrate flow at constant inlet flow and TMP were continuously determined. Flow direction is from the lower end to the upper end of the dialyzer and the filtration is performed in crossflow mode; the filtrate leaves the dialyzer at the upper lateral port.

To characterize the impact of protein adsorption on the filtration performance of dialyzer membranes, the recirculation setup displayed in Figure 2 was used. Milk (3 L, 3.5% fat, 37 °C) was used as a protein-containing and cell-free fluid, according to Ficheux et al. [42]. In line with human blood, milk consists of proteins of a broad molecular weight range, able to adhere to the dialyzer membrane. Previous comparisons between blood and milk showed the good applicability of milk for ultrafiltration experiments [42]. Pre- and post-dialyzer flow sensors allowed continuous monitoring of the inlet, outlet, and filtrate flow rates during the experiment. Two pumps were used to regulate the flows: the pump before the dialyzer regulated the inlet flow, whereas the pump downstream served as a regulator of the outlet flow and, therefore, also of the filtration flow. Pressure sensors before and after the dialyzer as well as on the filtrate side were used to determine the TMP over time according to the following formula:

(2) $$\mathrm{TMP}=\frac{Pressure\;pre\;dialyzer+Pressure\;post\;dialyzer}{2}-Pressure\;filtrate\;side$$

Two different experimental approaches were investigated: (1) determination of TMP increase over time (60 min and 240 min) at constant flow rates (inlet flow: 400 mL/min, outlet flow: 300 mL/min, filtrate flow: 100 mL/min) and (2) determination of filtrate flow reduction over time (240 min) at a constant inlet flow (400 mL/min) and constant TMP (75 mmHg). Each experiment for each dialyzer type was performed in triplicate.

### 2.4. Statistics

Mean ± SD (standard deviation) values are presented as summary statistic measures throughout the study. One-way ANOVA and Dunnett’s post hoc test were applied to compare the FX CorAL to the other dialyzers. The prerequisite of equal variances was tested using Levene’s test on a significance level of 0.05. All reported levels of significance (* *p* < 0.05, ** *p* < 0.01, *** *p* < 0.001) are given with regard to the FX CorAL dialyzer. Minitab^®^ 21.3 (Minitab, LLC, State College, PA, USA) was used for all statistical analyses.

## 3. Results

### 3.1. Impact of Protein Adsorption on Middle Molecule Clearance

**Figure 3:**
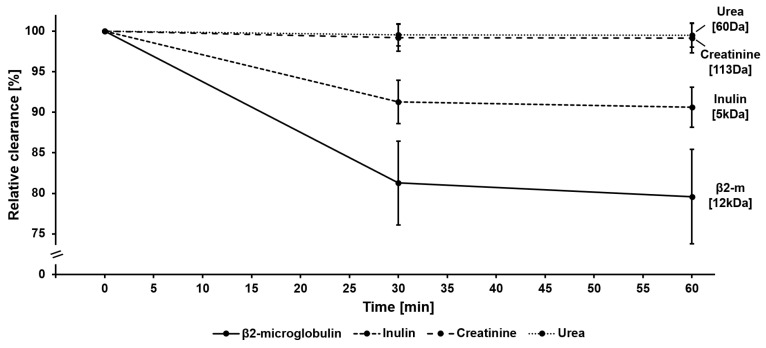
Reduction in β2-microglobulin, inulin, creatinine, and urea clearances after protein adsorption to the membrane. Clearances were measured before as well as after 30 min and 60 min plasma recirculation. Displayed is the mean ± SD clearance for all dialyzers tested relative to the initial measurement (before recirculation). Clearance of β2-microglobulin strongly reduced in the first 30 min and more slowly afterwards. The reduction in clearance depends on the molecular weight, with a lower reduction for inulin and only a little variation for creatinine and urea.

We first investigated how middle molecule clearance varies over time. The results of this experiment are summarized in Figure 3 and Table S1. Here, β2-clearance was measured at 0 min, as well as after 30 min and 60 min of protein fouling, induced by plasma recirculation. Mean β2-clearance across all dialyzers was 38.1 ± 8.4 mL/min/m^2^ at 0 min, 30.9 ± 6.9 mL/min/m^2^ at 30 min, and 30.2 ± 6.9 mL/min/m^2^ at 60 min. The relative clearance over time is depicted in Figure 3, showing a strong decrease in the β2-clearance in the first 30 min, which flattened during the next period (30–60 min). For comparison, clearance of the smaller-sized inulin decreased much less (0 min: 84.7 ± 8.0 mL/min/m^2^, 30 min: 77.3 ± 7.3 mL/min/m^2^, 60 min: 76.7 ± 7.1 mL/min/m^2^), and the clearances of the small molecules creatinine (0 min: 138.9 ± 4.1 mL/min/m^2^, 30 min: 137.8 ± 4.4 mL/min/m^2^, 60 min: 137.7 ± 4.0 mL/min/m^2^) and urea (0 min: 150.3 ± 4.4 mL/min/m^2^, 30 min: 149.7 ± 4.6 mL/min/m^2^, 60 min: 149.6 ± 4.5 mL/min/m^2^) varied little over the 60 min period.

In summary of these first experiments, the results confirmed previous findings that protein adsorption reduces the performance of dialysis membranes, especially in the first 30 min of treatment [11,12,17,19,24,25,26,27,28,29]. Moreover, the reduction in performance depends on the molecular weight of the respective molecule, as the adsorption of plasma proteins to the membrane has a stronger effect on larger proteins than on smaller solutes [24,25,26,27].

**Figure 4:**
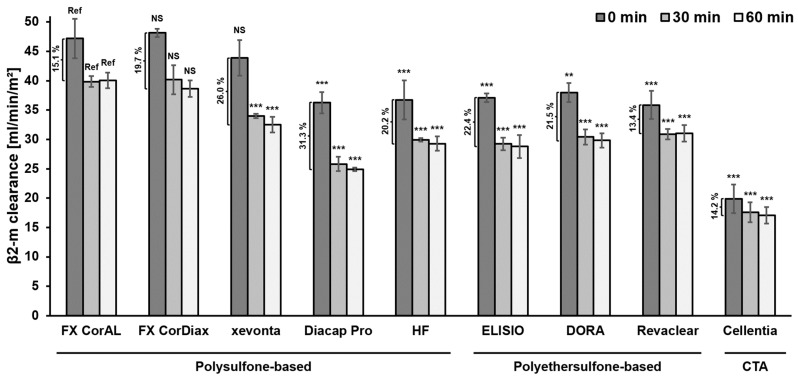
β2-microglobulin clearance across all tested polysulfone-, polyethersulfone-, and cellulose-based dialyzers at the three time points. Displayed is the mean ± SD β2-microglobulin clearance at 0 min (dark gray), 30 min (medium gray), and 60 min (light gray). The mean relative decrease in β2-microglobulin clearance between 0 min and 60 min is indicated for each dialyzer. Statistical significance is given with respect to the FX CorAL dialyzer. Ref: reference; NS: not statistically significant; CTA: cellulose triacetate; ** *p* < 0.01, *** *p* < 0.001. The FX CorAL dialyzer showed a low reduction in the initial β2-microglobulin clearance and had the highest clearance after 60 min of protein fouling.

In Figure 4, we compared middle molecule clearance across the different dialyzer types. Initial β2-microglobulin clearance (0 min) was highest for the three polysulfone-based dialyzers FX CorAL (47.2 ± 3.4 mL/min/m^2^), FX CorDiax (48.1 ± 0.7 mL/min/m^2^; *p* = 0.997 vs. FX CorAL) and xevonta (43.9 ± 3.0 mL/min/m^2^; *p* = 0.432) (Figure 4). All other polysulfone-, polyethersulfone-, and cellulose-based dialyzers had significantly lower initial β2-microglobulin clearance than the FX CorAL, with Diacap Pro (36.3 ± 1.8 mL/min/m^2^; *p* < 0.001; polysulfone-based), Revaclear (35.8 ± 2.4 mL/min/m^2^; *p* < 0.001; polyethersulfone-based), and Cellentia (19.9 ± 2.4 mL/min/m^2^; *p* < 0.001; cellulose-based) showing the lowest initial β2-microglobulin clearance. Across all dialyzers, the initial β2-microglobulin clearance decreased on average by 18.7% after 30 min of protein fouling and by a further 1.7% after an additional 30 min of protein fouling. The FX CorAL (15.1%), Revaclear (13.4%) and Cellentia (14.2%) showed the lowest decrease in β2-microglobulin clearance over 60 min, while Diacap Pro (31.3%), xevonta (26.0%), and ELISIO (22.4%) showed the highest decrease. Subsequently, after 60 min of protein fouling, the FX CorAL showed the highest β2-microglobulin clearance (40.0 ± 1.3 mL/min/m^2^), which was statistically significant in comparison to all other investigated dialyzers, except for the FX CorDiax dialyzer (38.6 ± 1.4 mL/min/m^2^; *p* = 0.716). Diacap Pro (24.9 ± 0.3 mL/min/m^2^; *p* < 0.001), ELISIO (28.8 ± 1.9 mL/min/m^2^; *p* < 0.001), and Cellentia (17.1 ± 1.4 mL/min/m^2^; *p* < 0.001) showed the lowest β2-clearance after 60 min of protein fouling across the polysulfone-, polyethersulfone-, and cellulose-based dialyzers, respectively.

In summary, these β2-microglobulin results support previous findings that the hydrophilic modification of the FX CorAL reduces protein adsorption to the membrane and stabilizes the performance over time [12,19,38]. Moreover, these experimental data are in line with previous clinical trials, which consistently showed the highest β2-microglobulin removal as compared to other polysulfone-, polyethersulfone-, or cellulose-based dialyzers [37,39,40,41].

### 3.2. Impact of Protein Adsorption on Filtration Performance

**Figure 5:**
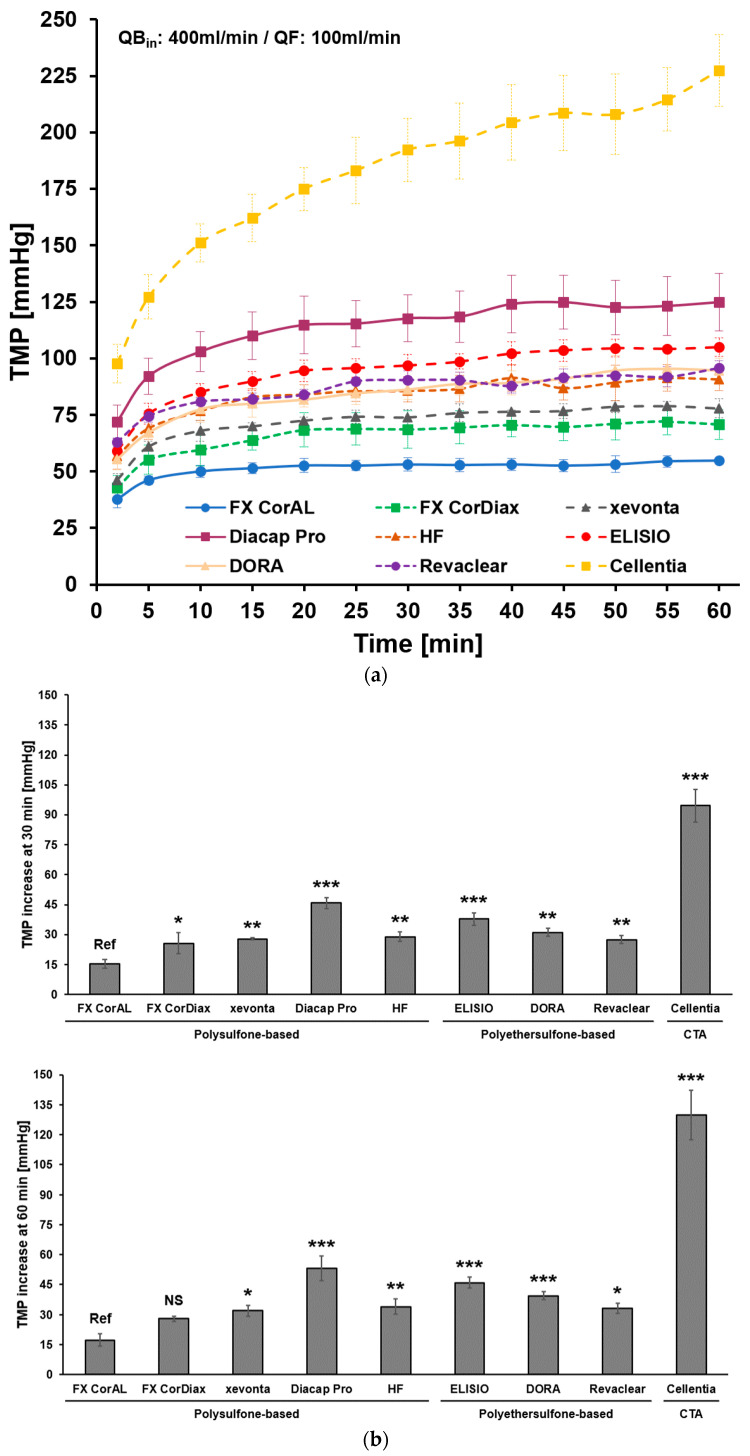
Determination of TMP increase over time: (**a**) In a recirculation experiment, the TMP was measured continuously at constant flow conditions (inlet flow: 400 mL/min, outlet flow: 300 mL/min, filtrate flow: 100 mL/min) for all tested polysulfone-, polyethersulfone-, and cellulose-based dialyzers. Over 60 min, mean TMP ± SD is displayed in 5 min intervals; (**b**) mean ± SD TMP increase at 30 min (upper panel) and 60 min (lower panel). Statistical significance is given with respect to the FX CorAL dialyzer. Ref: reference; NS: not statistically significant; CTA: cellulose triacetate; * *p* < 0.05, ** *p* < 0.01, *** *p* < 0.001. The FX CorAL dialyzer showed the lowest TMP increase after 30 min and 60 min of recirculation.

We next investigated the change in filtration characteristics of different dialyzer membranes over time. The results of this experiment are summarized in Figure 5. In this recirculation experiment, we determined the increase in the transmembrane pressure (TMP) at constant flow conditions (inlet flow: 400 mL/min, outlet flow: 300 mL/min, filtrate flow: 100 mL/min) over time (Figure 5a). In line with the previous experiment, TMP increased strongly during the first 30 min (37.2 ± 23.1 mmHg average increase across all dialyzers) and much slower during the next 30 min (additional 8.6 ± 10.2 mmHg average increase). When comparing the different dialyzer types, the FX CorAL showed the lowest TMP increase after 30 min (15.4 ± 2.1 mmHg increase vs. start), whereas Diacap Pro (45.8 ± 2.8 mmHg, *p* < 0.001), ELISIO (37.8 ± 3.2 mmHg, *p* < 0.001), and Cellentia (94.6 ± 8.1 mmHg, *p* < 0.001) showed the strongest TMP increase across the polysulfone-, polyethersulfone-, and cellulose-based dialyzers, respectively (Figure 5b). Also, after an additional 30 min (60 min total), the TMP increase was lowest for the FX CorAL dialyzer (17.3 ± 3.0 mmHg increase vs. start) and statistically lower compared to all other investigated dialyzers, except for the FX CorDiax dialyzer (27.9 ± 1.3 mmHg; *p* = 0.108).

In summary, to keep the filtrate flow constant, more pressure on the respective membranes is needed, as protein adsorption to the membrane increases the resistance to transport [11,12,19]. In line with the results obtained in Section 3.1, this increase in TMP mainly occurs in the first 30 min of recirculation. When comparing the different dialyzers, the FX CorAL showed the lowest increase in the TMP, in line with the lower protein adsorption by this hydrophilic membrane as characterized by previous studies [12,19,37,38].

**Figure 6:**
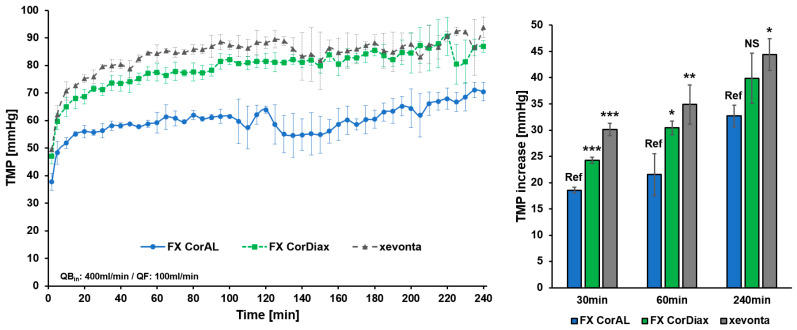
Determination of TMP increase over time. **Left** panel: In a recirculation experiment, the TMP at constant flow conditions (inlet flow: 400 mL/min, outlet flow: 300 mL/min, filtrate flow: 100 mL/min) was measured continuously for the polysulfone-based dialyzers FX CorAL, FX CorDiax, and xevonta. Over 240 min, the mean TMP ± SD is displayed in 5 min intervals. **Right** panel: mean ± SD TMP increase at 30 min (left), 60 min (middle), and 240 min (right). Statistical significance is given with respect to the FX CorAL dialyzer. Ref: reference; NS: not statistically significant; * *p* < 0.05, ** *p* < 0.01, *** *p* < 0.001. The FX CorAL dialyzer showed the lowest TMP increase after 30 min, 60 min, and 240 min of recirculation.

**Figure 7:**
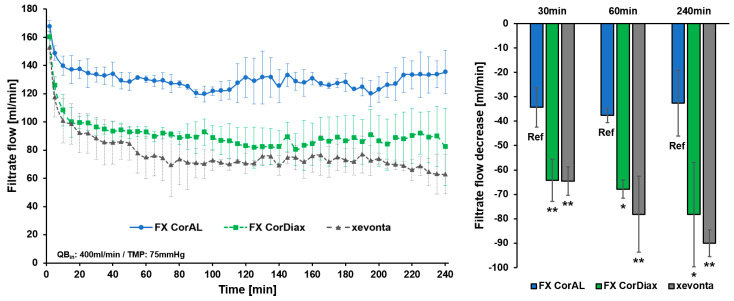
Determination of filtrate flow decrease over time. **Left** panel: In a recirculation experiment, the filtrate flow at a constant inlet flow (400 mL/min) and TMP (75 mmHg) was measured continuously for the polysulfone-based dialyzers FX CorAL, FX CorDiax, and xevonta. Over 240 min, the mean filtrate flow ± SD is displayed in 5 min intervals. **Right** panel: mean ± SD decrease in the filtrate flow at 30 min (left), 60 min (middle), and 240 min (right). Statistical significance is given with respect to the FX CorAL dialyzer. Ref: reference; * *p* < 0.05, ** *p* < 0.01. The FX CorAL dialyzer showed the lowest decrease in the filtrate flow after 30 min, 60 min, and 240 min of recirculation.

To additionally investigate the filtration characteristics of dialyzer membranes beyond the first hour of dialysis, we evaluated the three polysulfone-based dialyzers, namely FX CorAL, FX CorDiax, and xevonta, over a standard dialysis time of 4 h. The results of these experiments are summarized in Figure 6 and Figure 7.

In the first experiment, we again determined the increase in the TMP at constant flow conditions (inlet flow: 400 mL/min, outlet flow: 300 mL/min, filtrate flow: 100 mL/min) over time (Figure 6). Across all dialyzers, TMP increased on average by 24.3 ± 5.8 mmHg within the first 30 min of recirculation, by a further 4.6 ± 1.6 mmHg in the next 30 min, and by an additional 10.0 ± 1.0 mmHg in the time between 60 and 240 min (i.e., 1.7 mmHg increase per 30 min). When comparing the three dialyzers, the FX CorAL showed the lowest TMP increase at all three time points (vs. FX Cordiax: *p* < 0.001 [30 min], *p* = 0.028 [60 min], *p* = 0.078 [240 min]; vs. xevonta: *p* < 0.001 [30 min], *p* = 0.004 [60 min], *p* = 0.011 [240 min]).

In the second experiment, we measured the reduction in the filtrate flow at a constant inlet flow (400 mL/min) and constant TMP (75 mmHg) over 4h (Figure 7). In line with the previous experiments, filtrate flow decreased strongly within the first 30 min (54.4 ± 17.4 mL/min mean reduction across all dialyzers), while during the next 30 min of recirculation, filtrate flow decreased slowly by an additional 6.8 ± 5.9 mL/min and by a further 5.7 ± 9.4 mL/min in the time between 60 and 240 min (i.e., 1.0 mL/min per 30 min). Here, the FX CorAL again showed the lowest reduction in filtrate flows throughout the experiment (vs. FX CorDiax: *p* = 0.005 [30min], *p* = 0.014 [60 min], *p* = 0.017 [240 min]; vs. xevonta: *p* = 0.005 [30 min], *p* = 0.003 [60 min], *p* = 0.006 [240 min]).

In summary, these final experiments over 4 h supported the previous findings (Section 3.1 and Section 3.2) that the reduction in performance mainly occurs in the first 30 min. Protein fouling is a fast process, which leads to a reduction in performance in the first minutes of recirculation [11,12,19,24,25,26]. The FX CorAL dialyzer showed the most stable filtration characteristics over time, in line with previous data on low secondary membrane formation by its hydrophilic membrane [12,19,37,38].

## 4. Discussion

In the present in vitro study, we investigated the change in middle molecule clearance and filtration characteristics of different dialyzers over time. The results of this study show that the reduction in dialyzer performance occurs in the first minutes of exposure to a protein-containing solution and that the magnitude of the reduction is dependent on which membrane is used.

Patients with ESRD are a severely ill population with exceedingly high mortality rates [43,44,45,46,47,48,49,50,51]. The recently published CONVINCE trial provides hope, as patients treated with high-volume HDF experienced a significantly lower mortality rate than patients treated with conventional HD [8]. Such a survival benefit is suggested to be linked to the achieved convection volume as studies with lower delivered doses did not observe this positive effect [52,53]. Different underlying mechanisms for these benefits have been proposed, which include direct and indirect effects [1,11]. Among these, increased removal of uremic toxins, especially of middle molecules, has been discussed as the central element with the largest effect size and strength of evidence in the context of these benefits [1,11].

**Figure 8:**
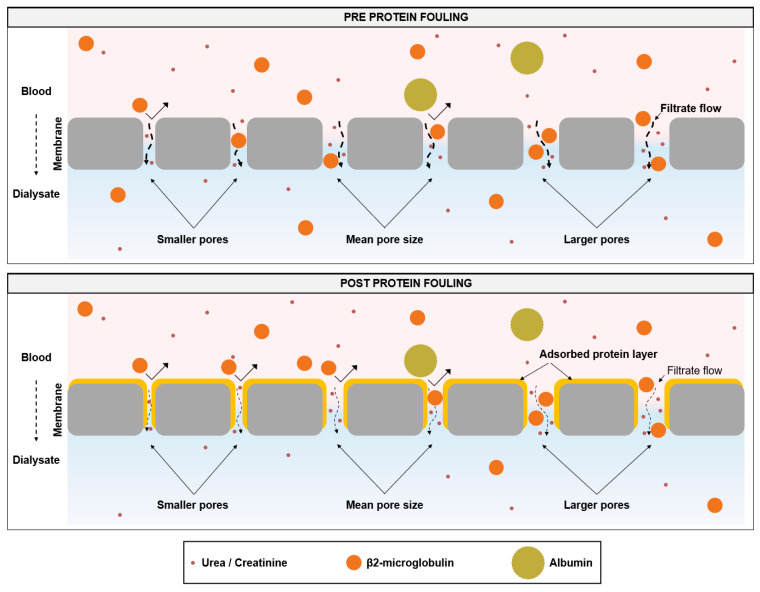
Schematic overview of the impact of protein adsorption on dialyzer membrane performance. The upper panel shows a membrane without a protein layer, allowing transport of molecules with higher molecular weight than with the membrane after protein fouling (yellow, lower panel). To achieve the same filtrate flow, the membrane in the lower panel would need a higher TMP; if the TMP is kept constant, the filtrate flow at the membrane after protein fouling would be lower.

To perform high-volume HDF that enables efficient removal of middle molecules, dialyzers are needed which support the achievement of treatment goals. The core element of the dialyzer is the membrane, which may differ substantially between dialyzers used in clinical practice. Membrane features, such as material and composition, geometry, or manufacturing, including the sterilization method, influence the initial membrane performance and its stability during treatment time [12,13,14,15,16,17,18,19]. In contrast to the early era of hemodialysis treatments with cellulose-based or synthetic low-flux membranes, advances in dialyzer technology have led to the development of synthetic high-flux membranes [11,14,15,54,55]. With these dialyzers, higher ultrafiltration rates are possible, allowing high-volume HDF treatments [11,56]. However, during dialysis treatment, the membrane flux reduces due to the adsorption of plasma protein to the membrane and the increased resistance to transport [11,12,17,19,24,25,26,27,28,29]. With these changed membrane characteristics during treatment, more pressure on the membrane is needed to achieve the same filtrate flow. Consistent with this concept, we observed a strong increase in the TMP at constant flow conditions, which was especially the case in the first 30 min of the experiment (Figure 5 and Figure 6). After this initial period, the TMP increased slightly during the next 30 min and even less between 60 and 240 min. Comparable results were observed for the clearance of middle molecules in this study. Β2-microglobulin clearance strongly reduced during the first 30 min and flattened afterward (Figure 3 and Figure 4). This kinetics in protein adsorption and performance reduction are consistent with previous findings. In 1986, Röckel et al. [24] tested the permeability of a polysulfone dialyzer in a hemofiltration mode among six patients. The authors found that during the first 10 min, the dialyzer was permeable to substances up to 66 kDa and dropped to less than 30 kDa within 20 min. The adsorption of plasma proteins to the membrane leads to a reduction in the effective pore size, which especially hinders the permeation of larger molecules, more than smaller solutes, such as urea or vitamin B12 [24,25,26,27] (Figure 8). Consistent with these previous findings, in the present study we found a strong reduction in β2-microglobulin clearance, whereas the clearance of the smaller-sized inulin (5 kDa) decreased much less and the clearances of the small solutes creatinine (113 Da) and urea (60 Da) remained nearly constant throughout the experiment (Figure 3).

**Figure 9:**
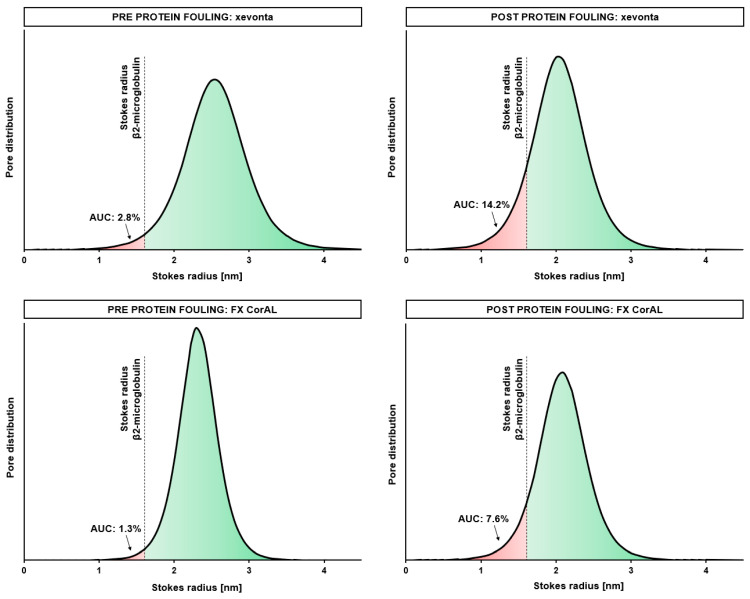
Effective pore radius before and after protein fouling. Data from Zawada et al. [19] was reanalyzed for the polysulfone-based dialyzers xevonta and FX CorAL, and the area, and the area under the curve (AUC) of the effective pore radius distribution was calculated for Stokes radii below (red) and above (green) the Stokes radius of β2-microglobulin.

In a previous experimental study, we investigated the changes in the albumin, myoglobin, and β2-microglobulin sieving coefficients in a plasma recirculation experiment over 240 min [19]. Sieving coefficients of all three proteins decreased, especially during the first 30 min and in higher magnitude for the larger proteins. This study also investigated the effective pore radius distribution before and after (120 min) protein adsorption and showed a clear reduction in the effective pore radii due to protein fouling. In Figure 9 we exemplarily reanalyzed data from this study for xevonta and FX CorAL to visualize the shift in the effective pore radius distribution as compared to the Stokes radius of β2-microglobulin. While before protein fouling most of the pores (97.2% for xevonta, 98.7% for FX CorAL) had an effective radius greater than the Stokes radius of β2-microglobulin, after protein fouling, the fraction was reduced to 85.8% (xevonta) and 92.4% (FX CorAL), respectively. This does not directly imply that the respective percentage of pores allow or block transport of β2-microglobulin through the pores, as other factors, such as membrane and molecule geometry, charge, or hydrodynamic conditions at the membrane are crucial in this context. Nonetheless, this gives a hint as to why protein adsorption to the membrane has a stronger impact on the clearance of larger molecules than of small solutes. Moreover, when comparing both dialyzers, these data support the findings from the present study regarding β2-microglobulin clearance, which showed higher values for the FX CorAL at the beginning and the end of the experiment (Figure 4). Therefore, for efficient removal of uremic toxins two main aspects are essential: (1) optimal initial pore size distribution and (2) low reduction in initial pore size during treatment due to protein fouling. Data from the previous study [19] show that the xevonta had a larger mean effective pore radius (2.54 nm) and pore radius range distribution (1.09 nm) at the beginning, as compared to the FX CorAL (mean effective pore radius: 2.31 nm, pore radius range distribution: 0.70 nm), leading to a higher percentage of pores with an effective radius below the Stokes radius of β2-microglobulin (and a higher percentage of pores with an effective radius above the Stokes radius of albumin) for xevonta. Moreover, after protein fouling, the mean effective pore radius decreased more strongly for the xevonta (decrease after 120 min: 0.50 nm), as compared to the FX CorAL (0.23 nm), supporting the performance stability of the FX CorAL dialyzer.

To sustain performance over treatment time, dialyzer membrane development focuses on reducing protein fouling. This is especially important in the case of HDF treatments, as the high ultrafiltration rates raise the transportation speed of plasma proteins to the inner membrane surface and subsequently increase the mass transfer resistance [11,57,58,59]. This additional barrier reduces the hydraulic permeability of the membrane and the elimination capacity of solutes, leading to lower achievable exchange volumes and removal capacity of uremic toxins. Moreover, the increased TMP to overcome this reduced membrane permeability may trigger alarms in the hemodialysis machine, which may lead to treatment interruptions and additional burden to clinic personnel [11,60].

To reduce protein fouling in order to stabilize performance and also to improve hemo/biocompatibility over treatment time, the latest membrane development introduced hydrophilic membrane modifications on the blood-facing surface [12,19,36,37,38,39,61]. To increase the hydrophilicity of synthetic polysulfone- or polyethersulfone-based membranes, the hydrophobic polymers are commonly blended with the hydrophilic polyvinylpyrrolidone (PVP). PVP is an inert agent and reduces protein adsorption to the membrane via the repulsive hydration force of the formed water layer [12,30,31,32,33,34,35,36]. The FX CorAL dialyzer contains such a hydrophilic membrane with increased PVP content on the blood-side surface and was found to induce low secondary membrane formation [12,19,36,37,38,39,61].

During standard hemodialysis membrane production, a spinning solution (consisting of core polymer, e.g., polysulfone, co-polymer (PVP), and solvent is run through the outer chamber of a spinneret, whereas the precipitation fluid is pumped in parallel through the inner orifice, leading to the formation of a hollow fiber [15]. For the FX CorAL, the spinning process was refined by adding additional PVP during the precipitation, and thus increasing the PVP content on the blood-side surface of the membrane. In a previous study, we compared the PVP content on the blood-side surface of the FX CorAL membrane to the predecessor membrane of the FX CorDiax by using X-ray photoelectron spectroscopy [36]. Here, we found that the FX CorAL membrane had a significantly higher PVP content on the blood-side surface than the FX CorDiax membrane (*p* < 0.05). Moreover, using contact angle measurements, we found that this PVP increase leads to a ~13% lower contact angle and, thus, higher hydrophilicity of the FX CorAL membrane as compared to the FX CorDiax membrane (*p* < 0.001) [12]. These data were confirmed by zeta-potential measurements, where we found that the FX CorAL membrane had the most neutral surface charge, as compared to the FX CorDiax (*p* < 0.001) and six further polysulfone-, polyethersulfone-, and cellulose-based dialyzers (*p* < 0.001) [38]. Finally, by investigating protein fouling by albumin sieving coefficient changes over time, the FX CorAL showed the lowest secondary membrane formation, which correlated with the membrane characteristics and hemocompatibility profile [38].

Results of the present study support these previous findings, as the FX CorAL showed both the highest β2-microglobulin clearance and a low reduction in the initial performance over time (Figure 4). Moreover, filtrate rates during recirculation at constant TMP decreased less as compared to all other investigated dialyzers (Figure 7). Vice versa, the increase in TMP at constant filtrate flows was less, pointing towards the lowest secondary membrane formation (Figure 5 and Figure 6).

These experimental studies are backed by four clinical studies (comPERFORM, eMPORA, eMPORA II, and eMPORA III) [37,39,40,41]. All studies investigated β2-microglobulin removal during HDF treatment as the primary outcome and compared the FX CorAL to other polysulfone-, polyethersulfone-, or cellulose-based dialyzers. In all these studies, with 253 patients in total, the FX CorAL consistently had the highest β2-microglobulin removal rates as compared to the 8 comparator dialyzers. Moreover, analyses of albumin sieving kinetics into the dialysate supported experimental findings of lower secondary membrane formation during treatment [37].

## 5. Conclusions

In conclusion, the results of the present study show that middle molecule clearance and filtration rate strongly decrease during the first minutes of dialysis. This study supports previous findings that protein adsorption to the dialyzer membrane reduces performance during treatment [11,12,17,19,24,25,26,27,28,29]. Hydrophilic membrane modification to reduce protein fouling, as implemented in the novel FX CorAL dialyzer, stabilizes performance over time. Future studies are needed to confirm whether the FX CorAL dialyzer may positively affect hemodiafiltration treatments by enabling more patients to achieve high-volume HDF.

## Figures and Tables

**Table 1:** Investigated dialyzers in the present study.

Dialyzer	Manufacturer	Membrane Name	Membrane Material	Sterilization	Surface [m^2^]
FX CorAL 80	Fresenius Medical Care	Helixone *hydro*	Polysulfone, polyvinylpyrrolidone	INLINE steam	1.8
FX CorDiax 80	Fresenius Medical Care	Helixone *plus*	Polysulfone, polyvinylpyrrolidone	INLINE steam	1.8
xevonta^®^ Hi 18	B. Braun	amembris polysulfone	Polysulfone, polyvinylpyrrolidone	Gamma	1.8
Diacap^®^ Pro 19H	B. Braun	α polysulfone pro	Polysulfone, polyvinylpyrrolidone	Gamma	1.9
HF18	Wego	N/A	Polysulfone-based *	Radiation *	1.8
ELISIO^TM^-17H	Nipro	Polynephron^TM^	Polyethersulfone, polyvinylpyrrolidone	Gamma	1.7
DORA^®^ B-18HF	Bain Medical Equipment	N/A	Polyethersulfone-based *	Radiation *	1.8
Revaclear 400	Baxter	Poracton	Polyarylethersulfone, polyvinylpyrrolidone	Steam	1.8
Cellentia^TM^ 17H	Nipro	N/A	Cellulose triacetate	Gamma	1.7

* Not further specified in the respective instructions for use or brochures; N/A: not available; note: FX CorAL 600 (1.6 m^2^), FX CorDiax 600 (1.6 m^2^), xevonta Hi 15 (1.5 m^2^), Diacap Pro 16H (1.6 m^2^), and ELISIO-15H (1.5 m^2^) were used for the filtration experiments.

## Data Availability

Data are contained within the article.

## Supplementary Materials

The following supporting information can be downloaded at: https://www.mdpi.com/article/10.3390/membranes14040083/s1, Table S1: β2-microglobulin, inulin, creatinine, and urea clearances after protein adsorption to the membrane.
