# Supplementary material for: Hydrophilic Modification of Dialysis Membranes Sustains Middle Molecule Removal and Filtration Characteristics
Source: Membranes (Basel). 2024 Apr 3;14(4):83. doi: 10.3390/membranes14040083 (PMC11052066; doi:10.3390/membranes14040083)
Supplement: Supplementary file 1 [file membranes-14-00083-s001.zip › membranes-2929448-supplementary.pdf]

Table S1. β2-microglobulin, inulin, creatinine, and urea clearances after protein adsorption to the membrane. Clearances were measured before as well as after 30 min and 60 min plasma recirculation. Displayed is the mean ± SD clearance for all dialyzers tested.

**Parameter**	**Clearance at 0 min [ml/min/m²]**	**Clearance at 30 min [ml/min/m²]**	**Clearance at 60 min [ml/min/m²]**
**β2-microglobulin [12 kDa]**	38.1 ± 8.4	30.9 ± 6.9	30.2 ± 6.9
**Inulin [5 kDa]**	84.7 ± 8.0	77.3 ± 7.3	76.7 ± 7.1
**Creatinine [113 Da]**	138.9 ± 4.1	137.8 ± 4.4	137.7 ± 4.0
**Urea [60 Da]**	150.3 ± 4.4	149.7 ± 4.6	149.6 ± 4.5
